# Ecosystem Health Assessment of Hulun Lake Using an eDNA Metabarcoding‐Based Phytoplankton Index of Biotic Integrity

**DOI:** 10.1002/ece3.74543

**Published:** 2026-10-27

**Authors:** Qi An, Ruiying Fu, Hao Cui, Yuan Wang, Yihan Huo, Yang Bai, Wei Ding, Bowen Xiao, Chengzhen Jia, Huamin Liu, Lixin Wang

**Affiliations:** ^1^ School of Ecology and Environment Inner Mongolia University Hohhot China; ^2^ Inner Mongolia Key Laboratory of River and Lake Ecology Hohhot China; ^3^ Ministry of Education Key Laboratory of Ecology and Resource Use of the Mongolian Plateau Hohhot China; ^4^ Ecological and Agricultural Meteorological Center of Inner Mongolia Autonomous Region Hohhot China

**Keywords:** ecological health assessment, environmental DNA‐metabarcoding, Hulun Lake, index of biotic integrity, phytoplankton

## Abstract

Hulun Lake, one of the largest freshwater lakes in China, is currently experiencing significant water quality deterioration. Therefore, water quality assessment is of paramount importance, as it not only provides critical insights into the current pollution status of lakes but also offers a scientific basis for effective lake management and ecological restoration. Using environmental DNA‐metabarcoding data from 13 sampling sites in Hulun Lake in June 2023, our study explored the spatial variation of α‐diversity, the community composition, and phytoplankton index of biotic integrity (P‐IBI). A total of 431 phytoplankton species were identified across 10 phyla, 191 families, and 335 genera, with Chlorophyta and Bacillariophyta predominating. Biodiversity was higher in the northern and southern regions, except for the site XHK, but lower in the central region. P‐IBI scores exhibited clear spatial variation, with relatively high scores in the central and northern regions, except for the site XHK, indicating comparatively favorable ecological conditions. In contrast, lower P‐IBI scores were observed in the southern region, except for DNHWZ and GZ, suggesting relatively greater ecological degradation. The P‐IBI scores were significantly negatively correlated with Water Quality Index (WQI) values, indicating that higher biotic integrity was generally associated with better physicochemical water quality. RDA and Spearman rank correlation analyses revealed that ammonia nitrogen (NH_3_–N) and total organic carbon (TOC) were positively correlated with P‐IBI, whereas temperature (T) and total nitrogen (TN) showed negative correlations with P‐IBI, indicating that spatial variation in P‐IBI was closely associated with nutrient status and organic carbon availability. Lake management should integrate both P‐IBI and WQI‐based assessment approaches, providing a scientifically robust basis for the identification of environmental pressures and the diagnosis of ecological health.

## Graphical abstract

Using environmental DNA metabarcoding data from 13 sampling sites, we assessed spatial variation in phytoplankton diversity, community composition, and biotic integrity across Hulun Lake. Phytoplankton diversity was higher in the northern and southern regions, whereas P‐IBI values indicated better ecological conditions in the central and northern regions and were significantly associated with WQI values and key environmental variables. Integrating P‐IBI with WQI provides a complementary framework for identifying environmental pressures and assessing the ecological health of Hulun Lake.

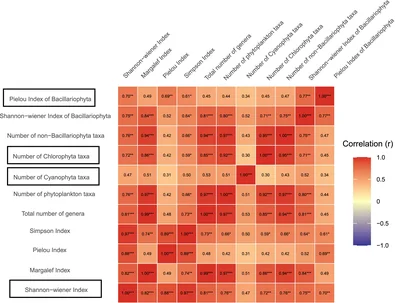

## 1 Introduction

Lakes serve as essential habitats for a wide variety of species and play a crucial role in providing diverse water resources, mitigating flood risks, supporting aquatic ecosystems, and conserving biodiversity (Lin et al. 2024; Xu et al. 2022). However, lake ecosystems are deteriorating at unprecedented rates in the Anthropocene (Zhang et al. 2023). Excessive water abstraction, eutrophication, pollution, and biological invasions have been widely recognized as major drivers of aquatic ecosystem degradation, threatening biodiversity and undermining ecosystem functioning and stability (Desta 2021). As a result, degradation of lake ecosystem has emerged as a critical global environmental and ecological issue that can no longer be overlooked (Yan et al. 2025).

Traditional water quality assessment methods typically classify water bodies by comparing measured physicochemical parameters with established water quality standards. In this framework, overall water quality is often determined by the poorest‐performing indicator (Kothari et al. 2021). Consequently, a water body may be classified as degraded even though most parameters comply with established water quality standards, resulting in an incomplete representation of its overall ecological condition. This approach does not fully capture the interactions and trade‐offs among different pollutants and is therefore limited in its ability to compare water quality across regions or time scales. The Water Quality Index (WQI), introduced in the 1960s, has been widely used as an integrated metric for evaluating water quality (Mo et al. 2024). It assigns weights to individual physicochemical parameters and aggregates multiple variables into a single composite value, thereby providing an overall indication of water quality status (Sanad et al. 2024). This approach is particularly well‐suited for water bodies characterized by high concentrations of organic matter (Hu et al. 2024). In addition, the WQI enables a graded and quantitative classification of water quality from Class I to inferior Class V, facilitating the identification of dominant pollution factors and their temporal dynamics, which supports water quality management and pollution control decision‐making (Wu et al. 2023). However, several studies have indicated that the WQI may not consistently correspond with changes observed in biological communities when assessing aquatic ecosystem health (Li et al. 2022), highlighting the need for integrated approaches that could reflect both environmental quality and ecological condition of aquatic ecosystems.

The Index of Biotic Integrity (IBI) is a multi‐metric framework that integrates diverse biological indicators and has been widely applied to assess the ecological health of aquatic ecosystems (Feng et al. 2021; Wu et al. 2012). The concept was first introduced by Karr (1981), who developed the fish‐based Index of Biotic Integrity (F‐IBI) for evaluating biological condition in stream ecosystems. However, the application of F‐IBI is considerably limited in river systems characterized by reduced flow regimes (Wang et al. 2022). In aquatic ecosystems, phytoplankton serve as primary producers that link physicochemical conditions with higher trophic levels and play a key role in regulating bottom‐up ecological processes (Zhu et al. 2021). Consequently, alterations in phytoplankton integrity can cascade through food webs and ultimately affect overall ecosystem structure and function. Compared with other aquatic organisms, phytoplankton are characterized by short generation times and rapid turnover rates, which enable them to respond sensitively to anthropogenic disturbances (Cabecinha et al. 2009; Domingues and Galvão 2007). Thus, P‐IBI enables a more accurate and comprehensive assessment of the health status of aquatic ecosystems and the extent of environmental disturbance they experience (Wu et al. 2012). This framework integrates multiple dimensions of ecological information, including temporal variations in physicochemical conditions, taxonomic composition, and functional group structure, and exhibits differential sensitivity to environmental stressors (Aura et al. 2020; Wu et al. 2012). The application of P‐IBI in the assessment of lake aquatic ecosystems has become a widely‐adopted approach (Baek et al. 2014; Cao et al. 2024; Wu et al. 2012).

Traditional studies related to phytoplankton communities relied primarily on microscopic identification. However, due to their tiny size and complex morphological characteristics, traditional microscopic identification is labor‐intensive, time‐consuming and requires expertise regarding morphological characteristic of different taxonomic group (Chen et al. 2024). In addition, the juvenile stages of closely related species within the same taxonomic group often show considerable morphological similarity (Haase et al. 2010). For example, juvenile copepods are difficult to distinguish based solely on morphological characteristics. Collectively, all these disadvantages limit the applicability and reliability of microscopic identification for species‐level classification and constrain its use in ecological studies requiring high‐frequency temporal monitoring, broad spatial coverage and efficient processing of large sample volumes. With the development of molecular ecology technique and high throughput sequencing, environmental DNA‐metabarcoding technique (eDNA‐metabarcoding) has emerged as a prospective tool for biodiversity associated studies. Compared with traditional survey methods, eDNA‐metabarcoding technique can reveal hidden taxonomic diversity, particularly for rare species that are difficult to capture, thus has been demonstrated to identify a greater number of species than conventional approaches (Lindeque et al. 2013). In addition, eDNA‐metabarcoding could effectively distinguish planktonic community compositions across different habitats while consistently maintaining high species detection rates (Chen et al. 2024). Furthermore, it can reduce overall sampling effort while enhancing detection efficiency, highlighting its broad potential for applications in ecological monitoring and environmental assessment (Valentini et al. 2016). Till now, this technique has been widely applied in a wide variety of research fields including ecological monitoring and ecological assessment of ecosystems (Leng et al. 2026). In addition, its applications have been extended to biomass estimation, water pollution source tracking (Rourke et al. 2022) and quantitative assessment of pollution levels across aquatic environments (Opog et al. 2024).

Hulun Lake (117°00′–117°41′ E, 48°30′–49°20′ N) is located in the northeastern part of the Inner Mongolia Autonomous Region, China (Wu et al. 2023). It is the largest lake in northern China (Zhang et al. 2018). Hulun Lake plays a critical role in regional climate regulation, water conservation, desertification control, and the maintenance of surrounding grassland ecosystem stability, serving as an important component of the ecological security barrier in northern China. However, in recent decades, Hulun Lake has experienced multiple and interacting environmental pressures, including increasing pollutant loads, lake shrinkage, wetland degradation, and localized grassland degradation and desertification (Hu et al. 2024). Nevertheless, its ecological health status remains poorly explored. To resolve this issue, the present study takes Hulun Lake in June 2023 as the research object and aims to investigate: (1) spatial variation in community characteristics and α‐diversity; (2) the spatial distribution patterns of the P‐IBI; and (3) the key environmental variables influencing the P‐IBI. The results can provide a scientific basis for subsequent management and ecological restoration in Hulun Lake.

## 2 Materials and Methods

### 2.1 Sample Collection and Processing

eDNA samples were collected from 13 sites across Hulun Lake in June 2023 (Figure 1). At each site, environmental variables were measured concurrently to characterize local water conditions. Water temperature (WT), pH, dissolved oxygen (DO), salinity (Sa), and conductivity (Cond) were measured in situ using a portable multiparameter water quality analyzer (HQ2200, Hach, Loveland, Colorado, USA). Secchi Disk depth (SD) was assessed with a Secchi disk (20 cm diameter, black‐and‐white pattern) under calm weather and diffuse light conditions. The remaining variables, including fluoride (F^−^), permanganate index (COD_Mn_), nitrate nitrogen (NO_3_^−^–N), total nitrogen (TN), ammonia nitrogen (NH_3_–N), chemical oxygen demand (COD_Cr_), five‐day biochemical oxygen demand (BOD_5_), total organic carbon (TOC), total phosphorus (TP), chlorophyll‐a (Chla), carbonate (CO_3_^2−^), bicarbonate (HCO_3_^−^), were analyzed in the laboratory within 24 h following sample collection. Detailed analytical methods, standards and detection limits are provided in Table S1.

**FIGURE 1:**
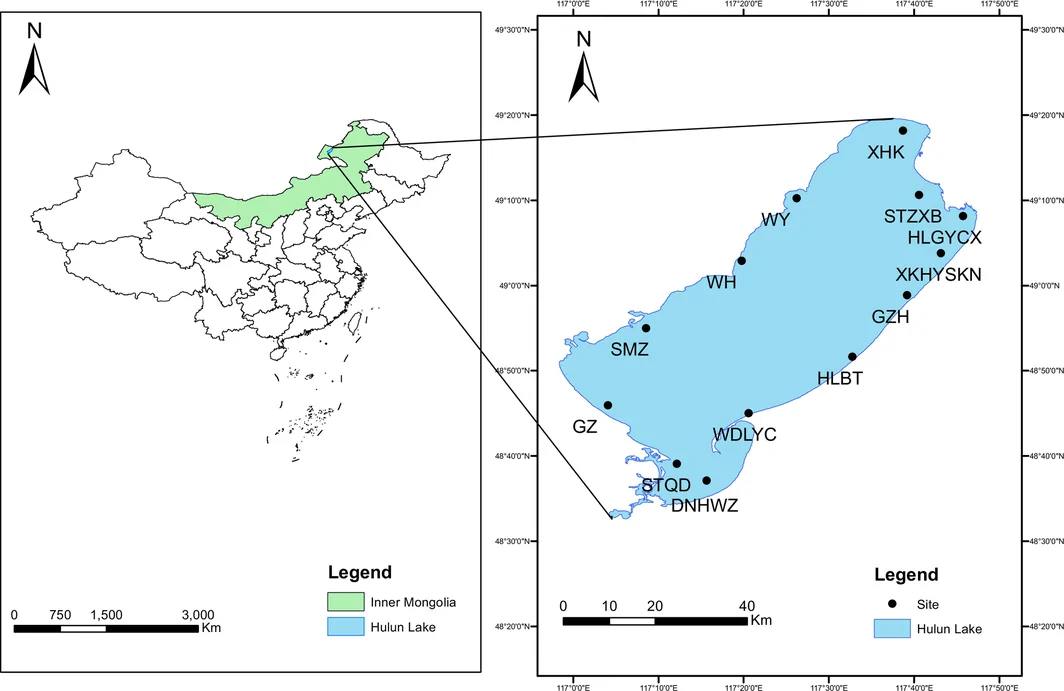
Study area and sampling site. The scale bar indicates distance in kilometers.

At each sampling site, 3 × 1 L surface water samples were collected as biological replicates using sterile bottles. Immediately after collection, the samples were transported in insulated containers and transferred to a refrigerator (4°C) within 3 h. Within 24 h of collection, each replicate was filtered independently through a 0.45 μm mixed cellulose ester (MCE) membrane filter (Millipore, USA) using a vacuum filtration system, yielding a total filtered volume of 3 L per site. The filters were individually preserved at −20°C until DNA extraction. eDNA was extracted separately from each filter using the cetyltrimethylammonium bromide (CTAB) method. The integrity of the extracted DNA was verified using 1.5% agarose gel electrophoresis, and its concentration and purity were quantified using a NanoDrop UltraC microvolume UV spectrophotometer (Thermo Fisher Scientific, Shanghai, China).

eDNA was amplified using primer sets targeting the V9 region of the 18S rRNA gene and the 16S rRNA gene, respectively. The eukaryotic 18S rRNA gene was amplified using primers 1389F (5′‐TTGTACACACCGCCC‐3′) and 1510R (5′‐CCTTCYGCAGGTTCACCTAC‐3′), whereas the prokaryotic 16S rRNA gene was amplified using primers 515F (5′‐GTGCCAGCMGCCGCGGTAA‐3′) and 806R (5′‐GGACTACHVGGGTWTCTAAT‐3′). PCR amplifications were carried out in 25 μL reaction volumes containing 50 ng of template DNA, 16 μM of each forward and reverse primer, 1× PCR buffer, 2.5 mM dNTPs, and 2 U of ExTaq DNA polymerase (Takara, Japan). A no‐template negative control was included in each amplification batch to detect potential contamination. PCR amplification was conducted under the following thermal cycling conditions: an initial denaturation at 95°C for 5 min; 35 cycles of denaturation at 95°C for 30 s, annealing at primer‐specific temperatures for 30 s, and extension at 72°C for 30 s; followed by a final extension at 72°C for 10 min. PCR products generated from biological replicates of each sampling site were pooled and purified using a SanPrep Column PCR Product Purification Kit (Sangon Biotech, Shanghai, China). Purified amplicons were sequenced on an Illumina HiSeq X Ten platform (Illumina, CA, USA) using paired‐end 150 bp (PE150) strategy.

### 2.2 Bioinformatic Analysis of eDNA Sequencing Data

Sequencing data processing was conducted following a standard amplicon analysis workflow. Initially, sequencing quality was evaluated using FastQC (v0.11.9, Babraham Institute, UK), after which adapter contamination and low‐quality sequences were removed with Trimmomatic (v0.39; Bolger et al. 2014). The filtered paired‐end reads were subsequently analyzed in QIIME2 (Bolyen et al. 2019). Within the QIIME2 framework, sample‐specific barcodes were used for read demultiplexing, and primer sequences were removed using the Cutadapt plugin. Error correction, denoising, and chimera removal were performed with DADA2 under default parameters, followed by the generation of amplicon sequence variants (ASVs). The resulting feature table and representative ASV sequences were retained as the basis for downstream taxonomic identification and ecological analyses.

Taxonomic annotation of ASVs was performed by comparing representative sequences against reference sequences in the GenBank 18S and 16S rRNA gene databases using BLASTN implemented in the SEED2 pipeline (Větrovský and Baldrian 2013). To improve annotation reliability, only alignments satisfying the predefined thresholds of ≥ 90% query coverage, ≥ 90% nucleotide identity, and an *E*‐value ≤ 10^−20^ were accepted. ASVs assigned to non‐target organisms were removed before ecological analyses, and the remaining phytoplankton‐specific ASV dataset was used for subsequent biodiversity assessment and community‐level analyses.

### 2.3 Water Quality Evaluation Methods

To comprehensively evaluate the water quality of Hulun Lake, a modified WQI developed by Liu (2022) was employed in this study.

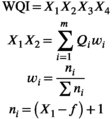

where *X*_1_ × *X*_2_ denotes the weighted average of single‐factor water quality pollution indices after weight adjustment, representing the overall pollution burden of the aquatic ecosystem. *X*_3_ refers to the number of parameters failing to meet the target water quality standards designated for the study area. *X*_4_ characterizes the discrepancy between the overall water quality class and the target class of the study area. *Q*_*i*_ represents the single‐factor water quality index of the *i*‐th water quality parameter. Moreover, m is the total number of parameters included in the WQI calculation and *w*_*i*_ is the corresponding weighting coefficient. The parameter *f* represents the predefined water quality target threshold, while *n*_*i*_ denotes the degree to which the *i*‐th parameter exceeds the applicable water quality standard. A lower WQI value indicates a better overall water quality condition (Wu et al. 2023).

### 2.4 Establishment and Application of an IBI

#### 2.4.1 Selection of Reference and Damaged Sites

Reference sites were identified by integrating relative water‐quality conditions within the current dataset with field‐based assessments of anthropogenic disturbance. Given the limited number of sampling sites (*n* = 13), approximately 25% of the sites were selected as reference sites to represent the relatively least‐disturbed conditions within Hulun Lake. Accordingly, the three sites with the lowest WQI values within the current dataset were initially identified, with the third‐lowest value used as an operational cutoff for reference sites selection. The ecological suitability of these candidate sites was then verified through field observations of anthropogenic disturbance. Specifically, reference sites showed no evident point‐source pollution, limited human activity along the shoreline, and minimal agricultural disturbance. Only sites satisfying both the WQI‐based screening and field‐based disturbance criteria were designated as reference sites, whereas the remaining sites were classified as damaged sites. Thus, the reference sites represent relatively least‐disturbed conditions within the study area rather than pristine or universally defined reference conditions.

#### 2.4.2 Determination of Candidate and Core Indicators

First, based on the indicators selected in Wan et al. (2021) and Zhang et al. (2019) for P‐IBI construction, we initially selected 14 candidate indicators for subsequent evaluation (Table 1).

**TABLE 1:** Selection of candidate indicators.

Indicator category	Indicator code	Candidate indicator	Change under disturbance
Species diversity	M1	Shannon‐wiener index	Decrease
	M2	Margalef index	Increase
	M3	Pielou index	Decrease
	M4	Simpson index	Increase
Community composition	M5	Number of phytoplankton phylum	Increase
	M6	Total number of genera	Increase
	M7	Number of phytoplankton taxa	Decrease
	M8	Number of Cyanophyta taxa	Decrease
	M9	Percentage of Cyanophyta taxa	Decrease
	M10	Number of Chlorophyta taxa	Decrease
	M11	Percentage of Chlorophyta taxa	Decrease
	M12	Number of non‐Bacillariophyta taxa	Increase
Pollution tolerance and characteristics	M13	Shannon‐wiener index of Bacillariophyta	Decrease
	M14	Pielou Index of Bacillariophyta	Decrease

The distribution characteristics of each candidate indicator were evaluated prior to screening. Indicators with excessively narrow or broad distribution ranges, excessively high standard deviations, zero values at more than 95% of the sampling sites, or non‐monotonic responses to increasing disturbance intensity were excluded from further analysis. The Indicator Quality (IQ) scoring method was employed to assess the discriminatory power of each indicator, following the Technical Guidelines for Monitoring and Assessment of Aquatic Ecosystems in Lakes and Reservoirs (Ministry of Ecology and Environment of China; HJ 1295–2023). Distributions between reference and damaged sites were compared using boxplots, and IQ scores were assigned based on the degree of interquartile range overlap. Only indicators with IQ ≥ 2 were retained for subsequent analysis (Ma et al. 2026).

To reduce redundancy within the final P‐IBI, Spearman's rank correlation analysis was conducted between pairs of the remaining indicators. When the absolute value of the correlation coefficient between two variables exceeded 0.75 (|*r*| > 0.75), a high degree of collinearity‐induced redundancy was inferred, and one of the two variables was subsequently excluded based on its relative ecological relevance and interpretability (Zhu et al. 2021).

#### 2.4.3 Calculation of P‐IBI

The selected indicators were standardized using the ratio method, which is widely regarded as an accurate and effective approach for dimensional normalization. Accordingly, core indicators were normalized based on their response direction to environmental stress. For parameters negatively associated with environmental stress, the 95th percentile of values across all sampling sites was used as the reference value, and scores were calculated as observed value divided by the reference value. For indicators positively associated with environmental stress, the 5th percentile was used as the reference value, and scores were computed as (maximum value − observed value)/(maximum value − reference value). The total P‐IBI score for each sampling site was obtained by summing the scores of all core indicators.

#### 2.4.4 Statistical Analysis and Visualization

Rarefaction curve analysis, dominant taxa analysis, and diversity index estimation were performed using the “vegan” package (Oksanen et al. 2025) in R 4.5.2 to assess sampling sufficiency, characterize phytoplankton community composition, and quantify community diversity. Prior to constrained ordination, variance inflation factor (VIF) analysis was conducted using the “car” package (Fox et al. 2001) to assess multicollinearity among environmental variables. Based on the June sampling dataset, VIF values were calculated for the environmental variables, and variables with VIF ≥ 10 were excluded while taking their ecological relevance into consideration. Detrended correspondence analysis (DCA) was subsequently performed using the “vegan” package to determine whether redundancy analysis (RDA) or canonical correspondence analysis (CCA) was more appropriate for examining the relationships between the P‐IBI core indicators and environmental variables. The appropriate constrained ordination approach was determined based on the gradient length of the first DCA axis. Prior to constrained ordination, the core indicators and environmental variables were standardized using Z‐score transformation. RDA was applied when the first‐axis length was < 3 standard deviation units, indicating a linear response of the community to environmental gradients; otherwise, CCA was considered more appropriate for longer gradients. Spearman's rank correlation analysis was performed using the “psych” package (Revelle 2026) to further assess associations among biological indicators. Least absolute deviations (LAD) regression was conducted in the “quantreg” package (Koenker 2025) to evaluate the relationship between P‐IBI and WQI while reducing the influence of potential outliers and deviations from normality. In addition, Pearson correlation analysis was performed using the “Hmisc” package (Harrell Jr 2003) to examine relationships among environmental variables. Variables exhibiting strong collinearity (correlation coefficients ≥ 0.75 in absolute value) were excluded before subsequent analyses. All visualizations were generated using the “ggplot2” package (Wickham 2011).

## 3 Results

### 3.1 Structural Characteristics of Phytoplankton Community in Hulun Lake

Rarefaction curves were used to assess the sufficiency of sequencing depth (Figure S1). The results showed that the rarefaction curves of phytoplankton species richness increased progressively with sequencing depth and eventually reached a plateau, indicating that the current sequencing depth was sufficient to capture the dominant species composition across all sampling sites in June. Following quality control, denoising, and chimera removal, 1,150,876 high‐quality reads were retained, yielding 3485 amplicon sequence variants (ASVs). A total of 431 species were detected, representing 10 phyla, 191 families, and 335 genera. The phytoplankton community was dominated by Chlorophyta, accounted for approximately 39.5% of the total read abundance, followed by Bacillariophyta (26.6%), Cryptophyta (12.9%), Cyanobacteriota (9.8%), Ochrophyta (8.9%), and Dinoflagellata (1.4%) (Figure 2). The remaining phyla, including Haptophyta, Streptophyta, Rhodophyta, and Glaucophyta, collectively accounted for less than 1% of the total abundance (Figure 2).

**FIGURE 2:**
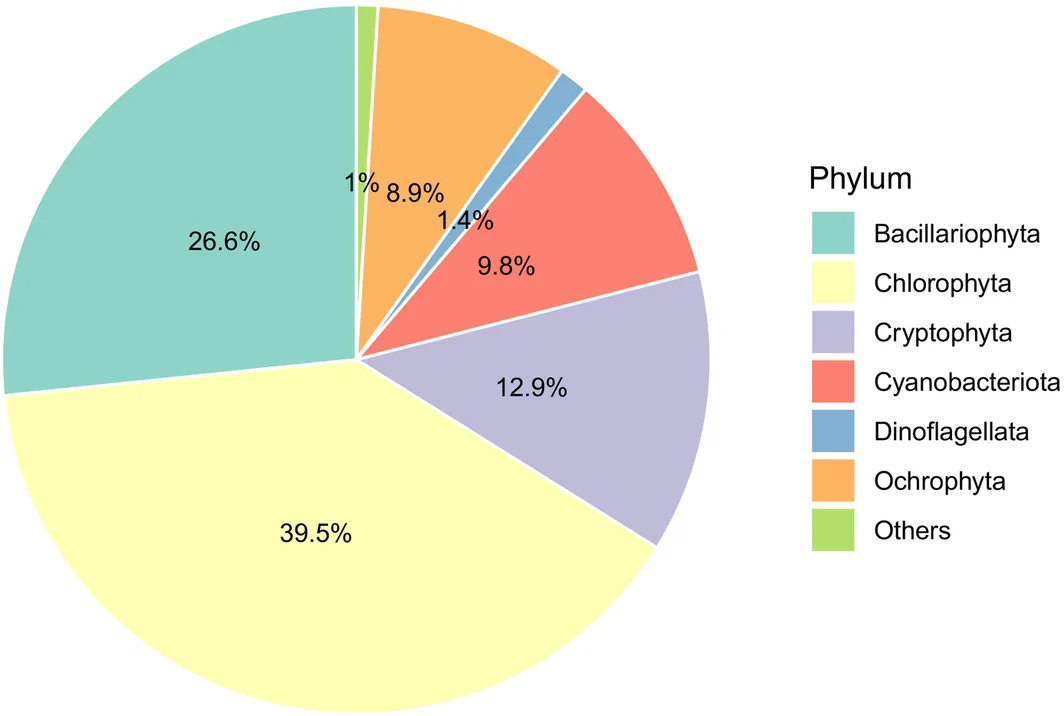
Phylum‐level composition and relative abundance of the phytoplankton community in Hulun Lake.

At the phylum level, the phytoplankton community was primarily composed of Chlorophyta and Bacillariophyta, although their relative contributions varied among regions (Figure 3a). The northern region was characterized by a relatively low abundance of Bacillariophyta. Compared with the northern and southern regions, the central region generally exhibited higher relative abundance of Bacillariophyta and Cyanobacteriota, accompanied by a lower proportion of Chlorophyta. In contrast, the southern sites were characterized by a higher contribution of Chlorophyta and a relatively low abundance of Cyanobacteriota.

**FIGURE 3:**
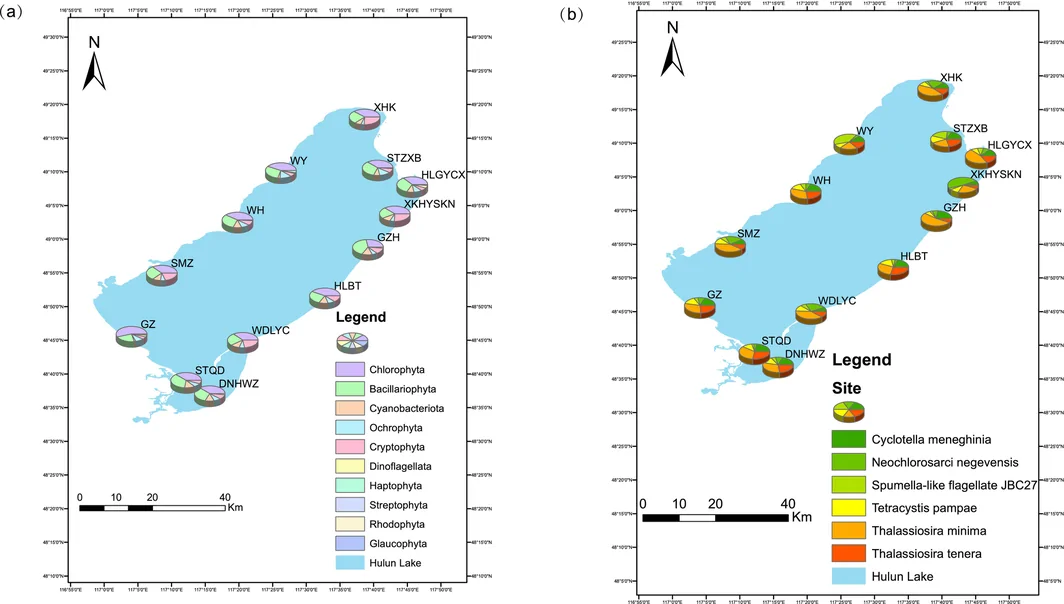
Spatial variations in the taxonomic composition and dominant species distribution of phytoplankton communities in Hulun Lake. (a) Relative abundance of phytoplankton phyla across sampling sites; (b) Spatial distribution patterns of dominant phytoplankton species.

The phytoplankton assemblage in Hulun Lake comprised seven dominant taxa, with dominance indices (*Y*) ranging from 0.021 to 0.109. The most dominant taxon was *Stephanodiscus* sp. KHR001 (*Y* = 0.109), followed by *Thalassiosira minima* (*Y* = 0.073), *Neochlorosarcina negevensis* (*Y* = 0.033), *Thalassiosira tenera* (*Y* = 0.032), *Cyclotella meneghinia* (*Y* = 0.030), *Tetracystis pampae* (*Y* = 0.022), and *Spumella*‐like flagellate JBC27 (*Y* = 0.021). Four dominant taxa were assigned to Bacillariophyta (*Stephanodiscus* sp. KHR001, *Thalassiosira minima* , *Thalassiosira tenera*, and *Cyclotella meneghinia*), whereas two belonged to Chlorophyta (*Neochlorosarci negevensis* and *Tetracystis pampae*). The remaining one dominant taxon, *Spumella*‐like flagellate JBC27, was assigned to Ochrophyta.

The relative contributions of dominant phytoplankton taxa varied among regions (Figure 3b). The northern region were generally characterized by higher proportions of *Neochlorosarcina negevensis* and *Spumella*‐like flagellate JBC27, whereas the central region exhibited relatively high proportions of *Thalassiosira minima* and *Cyclotella meneghinia*. In the southern region, *Thalassiosira tenera* and *Tetracystis pampae* accounted for relatively high proportions.

### 3.2 Spatial Distribution of α‐Diversity

In June 2023, phytoplankton species richness ranged from 166 to 329, with the lowest value recorded at HLBT and the highest at STZXB (Table S2). Richness at the phylum, family, and genus levels exhibited broadly consistent spatial patterns (Table S3). The Shannon, Simpson, and Pielou's evenness indices all reached their highest values at WY and their lowest values at XHK (Figure 4). Overall, these indices indicated lower diversity and evenness at the central region, whereas relatively higher values were observed in the northern and southern regions, with the exception of XHK.

**FIGURE 4:**
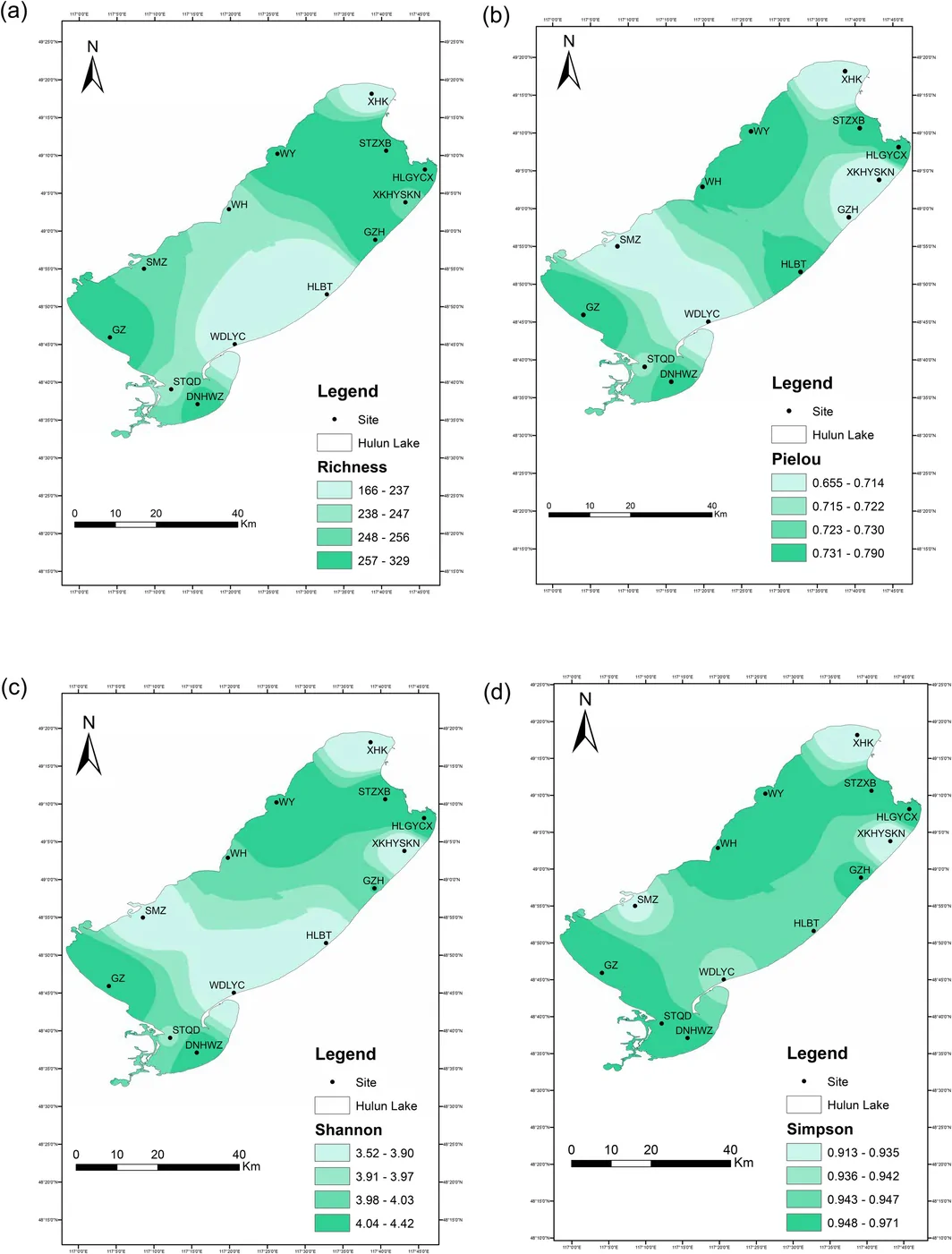
Spatial distribution of α‐diversity. (a) Richness, (b) Pielou Index, (c) Shannon‐Wiener Index, (d) Simpson Index. Increasing color intensity corresponds to increasing values of α‐diversity indices.

### 3.3 P‐IBI Evaluation System Construction

#### 3.3.1 Identification of Reference and Damaged Sections

Among all sampling sites, STZXB, HLGYCX, and WY exhibited the three lowest WQI values and also showed relatively low levels of anthropogenic disturbance based on field observations; these sites were therefore designated as reference sites (Figure S2). The remaining 10 sites (XHK, XKHYSKN, GZH, HLBT, WDLYC, DNHWZ, STQD, GZ, SMZ, and WH) were classified as damaged sites (Figure 5).

**FIGURE 5:**
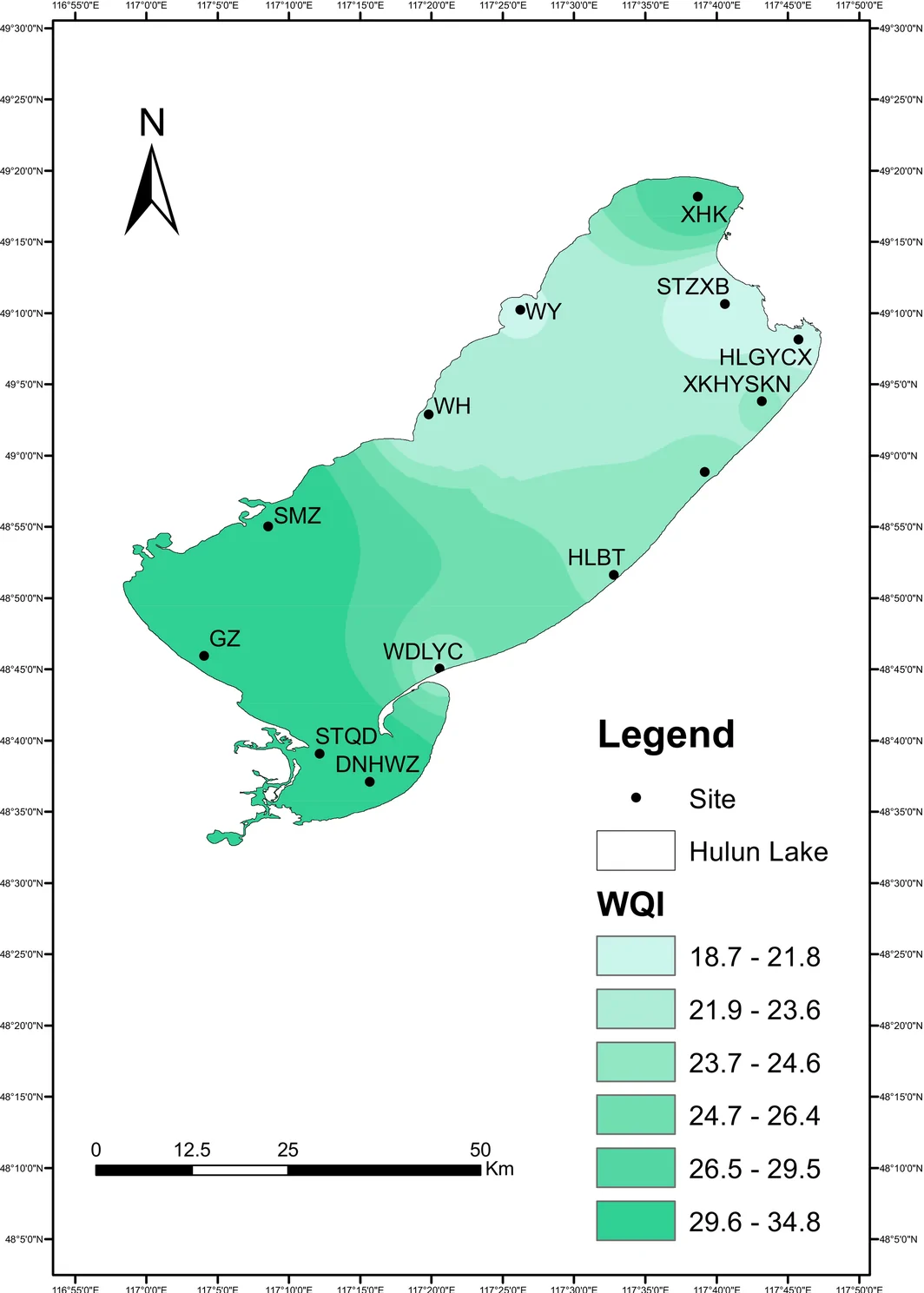
Spatial patterns and heterogeneity of the WQI in the study area. Increasing color intensity corresponds to higher WQI values and poorer water quality conditions.

#### 3.3.2 Discriminative Analysis of Candidate Indicators

Initially, the distribution range analysis showed that none of the candidate indicators had excessively narrow or broad distribution ranges, excessively high standard deviations, zero values at more than 95% of the sampling sites, or non‐monotonic responses to increasing disturbance intensity. Consequently, all candidate indicators were retained for subsequent evaluation. Discriminative analysis of the preliminarily selected candidate indicators between reference and damaged sites was conducted using boxplots. Indicator Quality (IQ) score was assigned to each of the candidate indicator. Eventually, three indicators, including number of phytoplankton phylum, percentage of Cyanophyta taxa and percentage of Chlorophyta taxa were removed for low IQ scores (Figure S3). The remaining 11 indicators with IQ ≥ 2 were retained for subsequent analysis (Figure 6).

**FIGURE 6:**
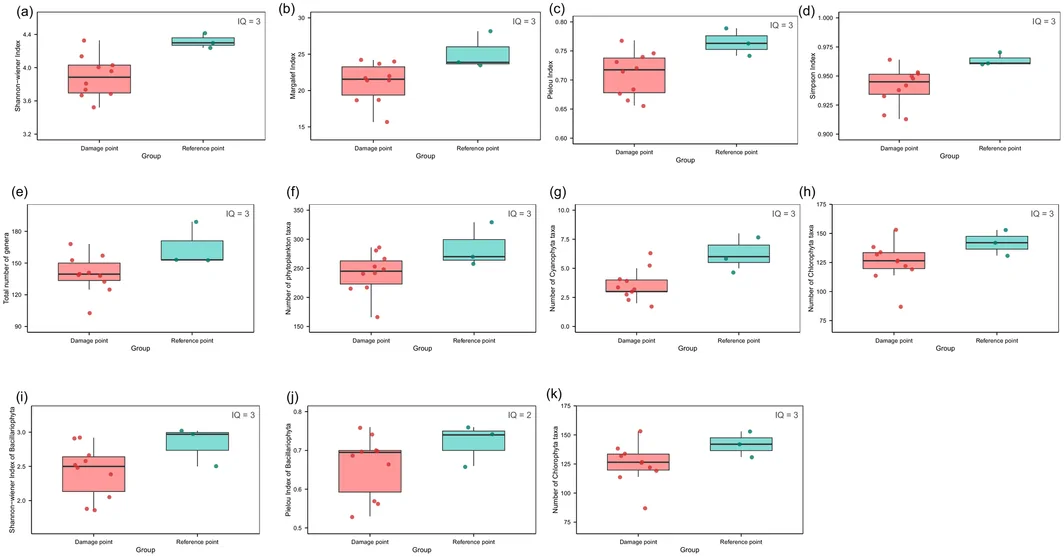
Boxplots of candidate indicators for the P‐IBI. Red indicates damaged sites, and blue denotes reference sites. Each indicator was assigned a corresponding IQ score based on the degree of separation between damaged and reference sites.

To remove redundance in core indicators, we conducted Spearman correlation analysis between pairs of the remaining 11 indicators. For pairs of indicators with an absolute correlation coefficient of |*r*| ≥ 0.75, one indicator from each pair was removed to reduce redundancy. Ultimately, Shannon‐wiener Index, number of Cyanophyta taxa, number of Chlorophyta taxa, and Pielou Index of Bacillariophyta were selected for P‐IBI assessment (Figure 7).

**FIGURE 7:**
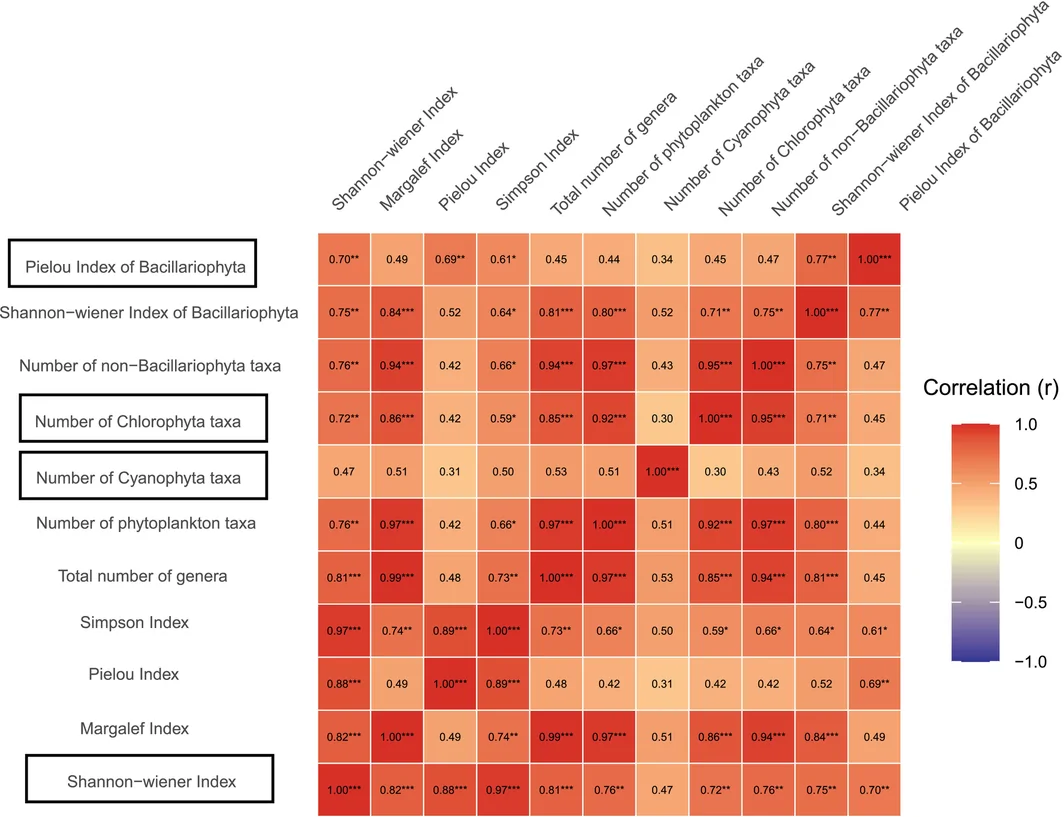
Spearman correlation analysis between pairs of candidate indicators following IQ screening. Orange and blue denote positive and negative correlations, respectively. Significant correlations are indicated by asterisks, with *, ** and *** representing *p* < 0.05, *p* < 0.01 and *p* < 0.001, respectively. The black boxes represent the final retained core indicators.

#### 3.3.3 P‐IBI Scoring

The four selected indicators were standardized using the ratio method. These four indicators, including M1 (Shannon‐wiener Index), M8 (number of Cyanophyta taxa), M10 (number of Chlorophyta taxa) as well as M14 (Pielou Index of Bacillariophyta) were negatively correlated with environmental stress (Table 2). The P‐IBI score was calculated as the sum of the four parameter scores (Figure 8).

**TABLE 2:** Calculation method for unifying the dimensions of P‐IBI core parameters.

Core indicators	Quantile	Reference value	Calculation formula	Change under disturbance
M1	95%	4.37	M1/4.37	Decrease
M8	95%	6.8	M8/6.8	Decrease
M10	95%	153	M10/153	Decrease
M14	95%	0.76	M14/0.76	Decrease

**FIGURE 8:**
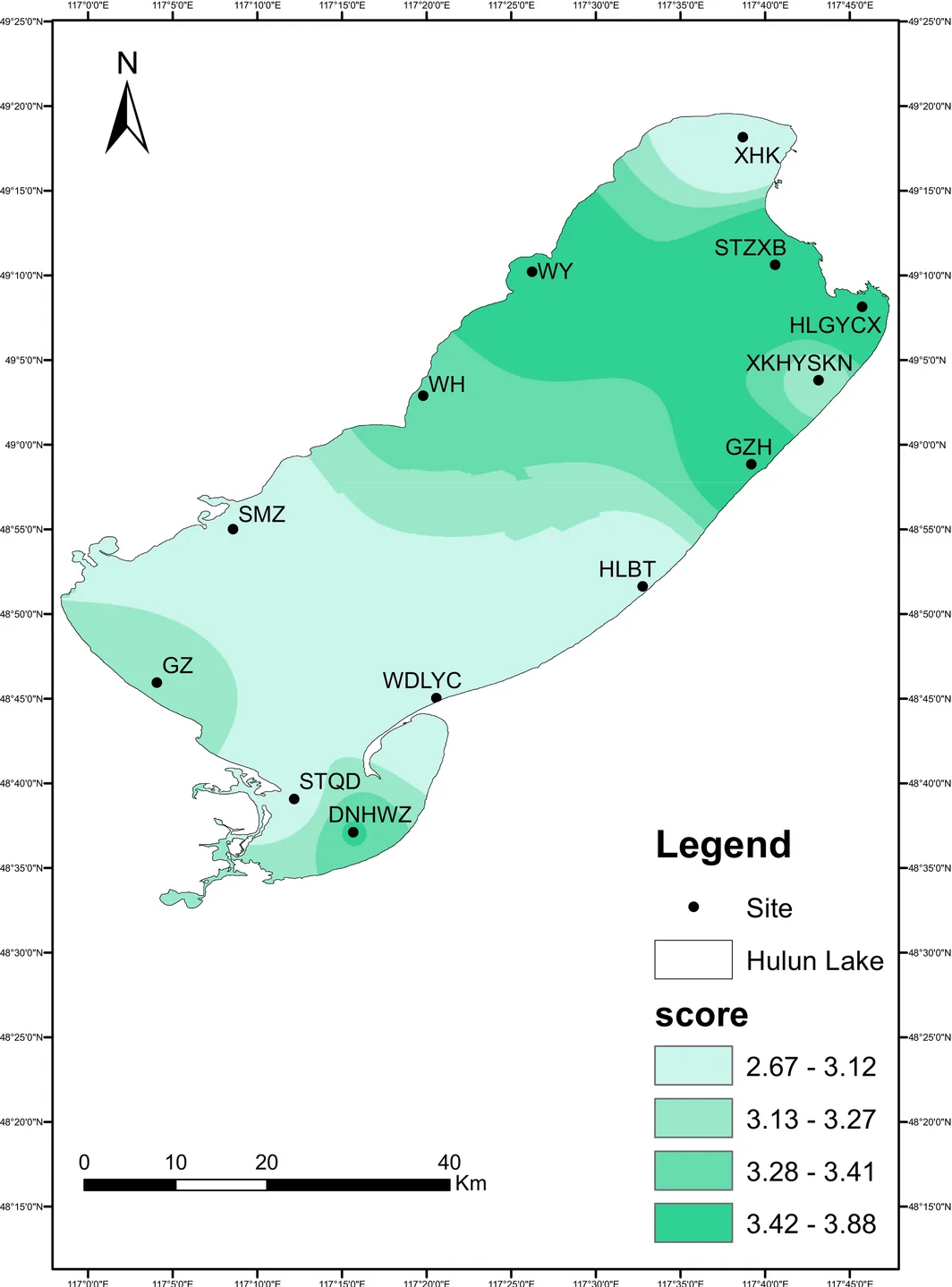
Spatial distribution of P‐IBI scores. Increasing color intensity corresponds to increasing P‐IBI scores.

The P‐IBI values of Hulun Lake ranged from 2.67 to 3.87, revealing pronounced spatial heterogeneity across the 13 sampling sites in June 2023 (Table S4). Higher P‐IBI scores were observed in the central and northern regions of the lake except for the site XHK, indicating relatively higher phytoplankton index of biotic integrity in these areas. In contrast, relatively lower P‐IBI scores were observed at sites in the southern region, except for DNHWZ and GZ. These spatial distribution patterns highlight the unevenness of biotic integrity across the lake and imply that local environmental conditions or anthropogenic pressures may differentially influence phytoplankton communities.

### 3.4 Relationship Between P‐IBI and WQI

LAD regression analysis revealed a significant negative association between P‐IBI and WQI. Since lower WQI values indicate better overall water environmental conditions, this significant relationship indicates a degree of consistency between biotic index‐based assessments and water quality‐based evaluations, suggesting that P‐IBI responds consistently to variations in water‐quality conditions and highlighting its applicability for assessing changes in aquatic environmental conditions (Figure 9).

**FIGURE 9:**
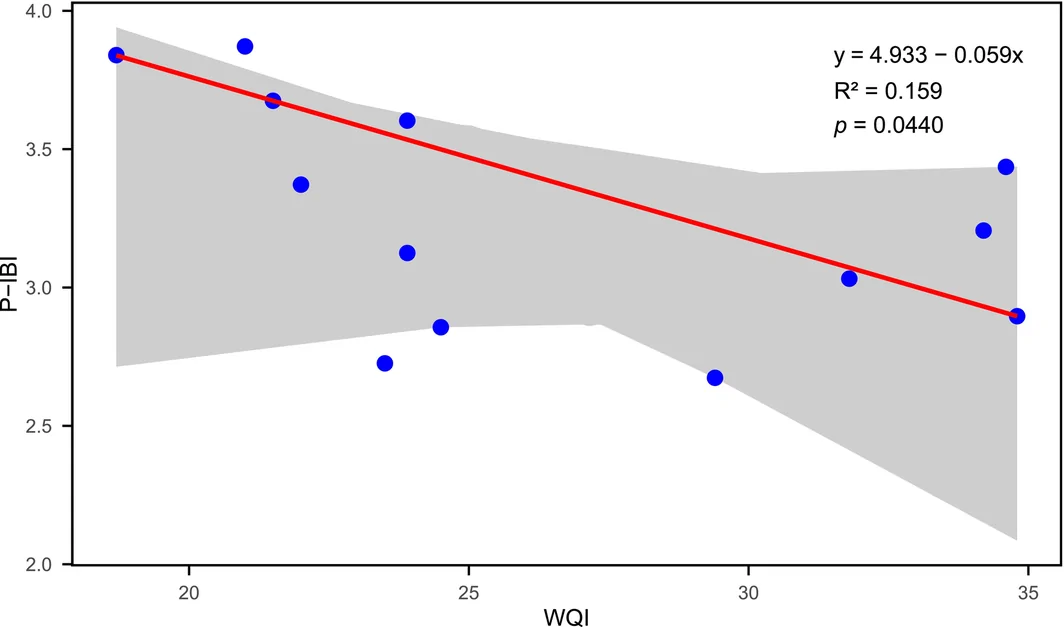
Relationship between P‐IBI and WQI assessed using least absolute deviations (LAD) linear regression. The solid red line represents the fitted LAD regression line, while the gray shaded area denotes the corresponding 95% confidence interval.

### 3.5 Relationship Between P‐IBI and Environmental Variables

DCA indicated that the gradient length of the first axis was less than 3.0, suggesting that RDA was appropriate for subsequent analyses (Table S5). To reduce multicollinearity, Pearson correlations were first calculated, and variables with strong pairwise correlations were excluded based on ecological relevance (Figure 10). Considering the ecological effects, 10 variables were ultimately retained, including T, COND, NH_3_–N, NO_3_^−^–N, COD_Mn_, COD, TN, TOC, turbidity, and CO_3_^2−^.

**FIGURE 10:**
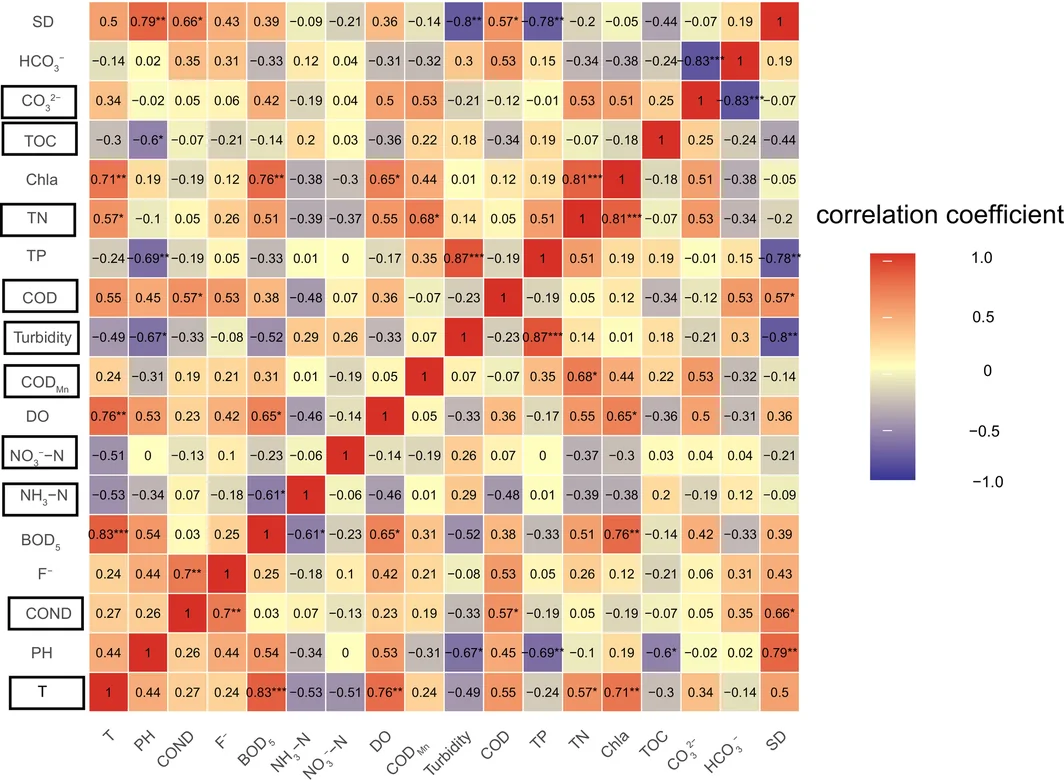
Heatmap of Pearson correlation Analysis for all Environmental variables. Orange and blue denote positive and negative correlations, respectively. Significant correlations are indicated by asterisks, with *, ** and *** representing *p* < 0.05, *p* < 0.01 and *p* < 0.001, respectively. The black boxes represent the final retained core indicators.

Based on the VIF results and ecological relevance, five environmental variables were retained for subsequent analysis, including NH_3_–N, NO_3_^−^–N, TOC, TN, and T. RDA results indicated that the first two ordination axes explained 33.6% of the variation in phytoplankton core indicators and environmental variables (Figure 11a). NH_3_–N and TOC were positively associated with P‐IBI, with TOC exhibiting the stronger relationship. Conversely, T and TN were negatively associated with P‐IBI. In contrast, elevated temperature and nitrogen concentrations, were associated with a decline in P‐IBI. In contrast, M1 (Shannon‐wiener Index) and M14 (Pielou Index of Bacillariophyta) were generally positively associated with TOC and NH_3_–N. M1, M8, and M10 were significantly positively correlated with P‐IBI. TN showed a significant negative association with M8, whereas TOC was positively associated with M8. In contrast, NH_3_–N and NO_3_^−^–N exhibited a positive association with M10 (the number of Chlorophyta taxa), while T was significantly negatively associated with the same indicator. For the June 2023 samples, Spearman correlation analysis further characterized the relationships among environmental variables, core indicators, and P‐IBI (Figure 11b). Overall, the correlation patterns were highly consistent with those revealed by the RDA, further supporting the robustness of the identified relationships between environmental variables and core indicators.

**FIGURE 11:**
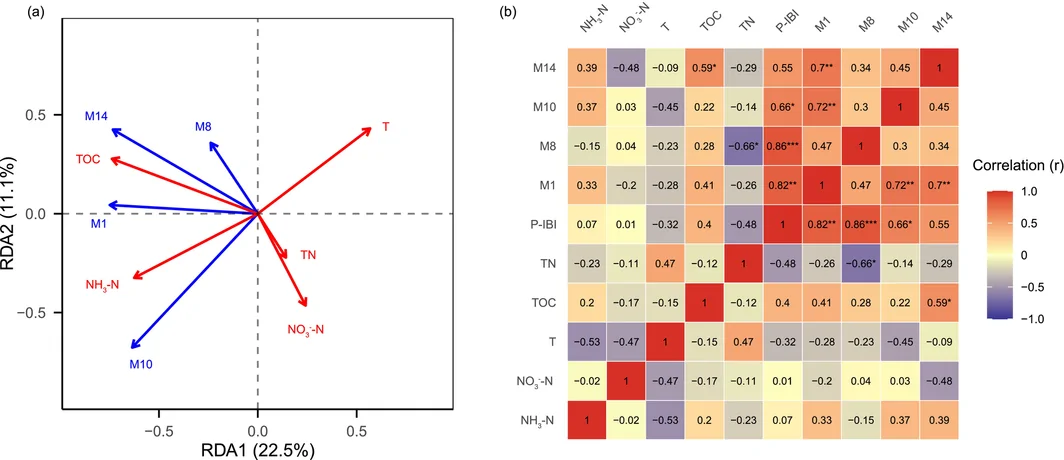
Multivariate and pairwise relationships between environmental variables, core indicators, and P‐IBI scores. (a) RDA depicting the integrated effects of environmental variables on core indicators and P‐IBI; (b) Spearman rank correlation analysis and pairwise scatter plots illustrating the relationships among environmental variables, P‐IBI, and core indicators. Significant correlations are indicated by asterisks, with *, **, and *** representing *p* < 0.05, *p* < 0.01, and *p* < 0.001, respectively.

## 4 Discussion

### 4.1 Community Structure of Phytoplankton

In June 2023, a total of 431 phytoplankton species, belonging to 335 genera and 10 phyla, were recorded across all sampling sites. In comparison, only 57 and 96 species were identified in June 2018 (Li et al. 2021) and in July to August 2021 (Hu et al. 2026), respectively. Overall, species abundance in June 2023 was markedly higher than in 2018 and 2021, which may partly reflect methodological differences. Reliance on traditional morphological identification is likely to underestimate actual species richness, as this approach primarily depends on the microscopic examination of morphological traits. Small‐sized or morphologically similar taxa are often difficult to distinguish, which may lead to misidentification or taxonomic merging. Moreover, low‐abundance or seasonally occurring rare species may remain undetected due to limited sampling probability, further affecting estimates of total species richness. In addition, morphological identification is highly dependent on the expertise of the observer, and subjective differences among researchers can compromise comparability of results. Conversely, molecular approaches such as eDNA metabarcoding generally recover far more taxa than microscopy, a pattern consistently reported for lakes worldwide (Brown et al. 2022; Hanžek et al. 2024). Consequently, part of the disparity in species richness between 2023 and earlier years likely stems from methodological differences, and should therefore not be interpreted as evidence of a true increase in phytoplankton biodiversity. However, it should be noted that α‐diversity obtained using the eDNA metabarcoding approach may be influenced by residual DNA originating from organisms in surrounding inflowing rivers, which can lead to an overestimation of species richness. Such bias introduced by exogenous DNA inputs represents a potential limitation of this method that should be carefully considered. Moreover, estimates of species richness derived from metabarcoding are inherently influenced by the choice of molecular marker, the completeness and taxonomic resolution of reference databases, and bioinformatic processing parameters, including sequence‐filtering and taxonomic‐assignment thresholds. In addition, metabarcoding data remain inherently semi‐quantitative and may not accurately reflect the absolute abundance or occurrence probability of individual taxa (Ruppert et al. 2019). Therefore, the higher species richness observed in June 2023 should be interpreted primarily as a more comprehensive representation of the molecularly detectable species pool, rather than as direct evidence of a genuine increase in phytoplankton diversity relative to 2018 and 2021.

In June 2023, Chlorophyta and Bacillariophyta dominated the phytoplankton assemblage, consistent with the community patterns reported in 2018 (Li et al. 2021) and 2021 (Hu et al. 2026). The recurrent dominance of these two phyla across surveys suggests that the broad taxonomic structure of the phytoplankton community has remained relatively stable at the phylum level. Comparable dominance patterns have also been documented during the ecological recovery of shallow lakes, particularly following reductions in cyanobacterial dominance (Zheng et al. 2026). Such temporal consistency may reflect persistent environmental filtering in Hulun Lake, favoring taxa that are well adapted to its prevailing local environmental conditions. In addition, species turnover within these dominant phyla may buffer ecosystem functioning when functionally similar taxa replace one another in response to environmental change (Kim et al. 2020). Therefore, despite potential interannual variation at finer taxonomic levels, the persistent dominance of Chlorophyta and Bacillariophyta indicates a degree of broad‐scale compositional stability in the phytoplankton community and may contribute to the functional stability of the Hulun Lake ecosystem.

### 4.2 Environmental Variables Influencing P‐IBI

Environmental variables are primary drivers of phytoplankton community composition across aquatic ecosystems, ultimately shaping variations in P‐IBI scores (Long et al. 2026). Serving both as an indicator of phytoplankton physiological status and as a source of carbon and nutrients, TOC can, within an optimal concentration range, stimulate phytoplankton growth and metabolism, thereby sustaining or enhancing the biological integrity of the community (Martens et al. 2024). The ecological effects of inorganic nitrogen are strongly contingent on nitrogen form and the broader environmental context. Consistent with this interpretation, P‐IBI scores were positively correlated with NH_3_–N, suggesting that moderate ammonium availability may support phytoplankton growth and community integrity. Elevated water temperature or increased TN often exacerbate eutrophication and alter aquatic conditions. In particular, TN enrichment can promote the proliferation of dominant phytoplankton species (Shetye et al. 2019), which may reduce community diversity and structural stability, resulting in an inverse relationship between T and TN with P‐IBI. These findings are consistent with a lake mesocosm warming experiment, which showed that the interactive effects of warming and nitrogen enrichment led to the lowest phytoplankton diversity, highlighting the potentially synergistic effects of climate warming and nutrient enrichment on phytoplankton community structure (Dashkova et al. 2022). Shannon‐wiener Index, Number of Chlorophyta taxa, and Pielou Index of Bacillariophyta were positively correlated with TOC and NH_3_–N, suggesting that the coupled availability of organic carbon and inorganic nitrogen may, to some extent, promote phytoplankton diversity and community structural evenness. Ammonium nitrogen represents an energetically favorable nitrogen source because its assimilation bypasses the reductive steps required for nitrate utilization. Consequently, fast‐growing Chlorophyta often preferentially assimilate ammonium, thereby gaining a metabolic advantage under conditions of elevated ammonium availability (Glibert et al. 2016). Consistent with our findings, a mesocosm experiment in a temperate eutrophic lake demonstrated that Dissolved Organic Carbon (DOC) enrichment favored diatoms, particularly in benthic compartments, likely by stimulating sediment biogeochemical processes that enhanced the availability of silicate and other nutrients essential for frustule formation (Fonseca et al. 2022). Accordingly, the moderate concentrations of NH_3_‐N and TOC currently recorded in Hulun Lake in June 2023 may fall within a favorable range that supports the coexistence and sustained dominance of Bacillariophyta and Chlorophyta. This interpretation is consistent with recent surveys of Hulun Lake, which have consistently reported phytoplankton communities dominated by Bacillariophyta and Chlorophyta (Zhang et al. 2025).

### 4.3 Implications for Lake Management Based on WQI and P‐IBI

P‐IBI showed a good fit with WQI, and both assessment approaches exhibited similar spatial patterns, with generally better conditions in the central and northern regions and poorer conditions in the south. This spatial concordance suggests that P‐IBI responds effectively to environmental stressors associated with physicochemical deterioration and captures spatial variation in the ecological quality of Hulun Lake. The observed pattern may reflect the combined influence of aquatic environment and anthropogenic disturbance (Qin et al. 2023). The lower P‐IBI scores in the southern region, which are closer to residential areas and pastures, may be associated with multiple pressures, including agricultural non‐point source pollution, livestock grazing, and pollutant inputs from inflowing rivers. In contrast, the central and northern regions are characterized by more open‐water habitats, lower human disturbance, and better habitat quality, thereby supporting higher biotic integrity.

The spatial pattern observed in June 2023 was broadly consistent with previous applications of P‐IBI in other lake ecosystems. Studies in the Taihu Basin likewise reported strong spatial concordance between P‐IBI assessments of biotic integrity and WQI‐based evaluations of water quality, despite differences in WQI formulation and the interpretation of index values among studies (Wu et al. 2019). Thus, the good agreement and similar spatial patterns between the P‐IBI ecological assessment and the WQI‐based water quality assessment observed in Hulun Lake are unlikely to be isolated phenomena, but may instead reflect a broader pathway linking environmental stress, physicochemical changes, and biological community responses in freshwater ecosystems. The spatial concordance between the two indices further indicates that integrating biological and physicochemical indicators can reveal complementary dimensions of lake ecosystem condition and provide a more comprehensive assessment of ecological quality than either approach alone (Wu et al. 2019). This consistency also highlights the potential of P‐IBI for identifying disturbed areas and ecological degradation in Hulun Lake, thereby providing a useful basis for pollution control, ecological restoration, and continued monitoring in the southern region.

Based on the consistency of spatial distribution patterns between P‐IBI scores and WQI values in June 2023, a spatially differentiated and finely targeted management strategy should be implemented for water environment management in Hulun Lake. The integration of these two approaches provides complementary and mutually validating evidence for identifying environmental pressures and diagnosing aquatic ecosystem health. Priority should be given to strengthening pollution source control and ecological restoration in the southern region, which might be significantly affected by agricultural non‐point source pollution, livestock activities, and pollutant inputs from inflowing rivers. In contrast, the central and northern regions exhibit relatively better ecological conditions and should therefore be managed primarily for conservation, with strict limitations on anthropogenic disturbances. The establishment of an integrated management framework combining pollution control, ecological restoration, and spatially differentiated protection can provide strong support for the overall improvement and long‐term stable recovery of the Hulun Lake aquatic ecosystem.

### 4.4 Limitations

In this study, environmental DNA‐metabarcoding was used for species detection. Compared with conventional microscopic identification, eDNA‐based approaches generally provide greater detection sensitivity and are particularly effective for detecting rare, small‐bodied, or morphologically cryptic taxa, thereby potentially recovering a greater number of species. However, eDNA detections do not necessarily correspond directly to the living phytoplankton assemblage present at the time of sampling. DNA released from dead organisms, resting stages, or allochthonous material transported by water currents may persist in the aquatic environment and broaden the apparent range of detected taxa. In addition, methodological factors, including variation in DNA extraction efficiency, primer bias, differential amplification efficiency, and the completeness and taxonomic resolution of reference databases, may result in false negatives, taxonomic misassignments, or uneven detection probabilities among different taxonomic groups. Therefore, although eDNA approaches substantially improve the sensitivity and taxonomic coverage of species detection, the resulting assemblage cannot be regarded as a complete representation of the actual taxonomic composition and community structure of living phytoplankton. Future studies should integrate eDNA metabarcoding with conventional microscopic identification to improve the robustness of species identification and enhance the reliability of phytoplankton community characterization and ecological assessment.

Furthermore, this study was based on a single phytoplankton survey and sampling campaign conducted in June 2023. Accordingly, the ecological health assessment derived from the P‐IBI primarily reflects the condition of Hulun Lake during this specific sampling period and should not be interpreted as representative of its year‐round ecological status. Nor can it capture seasonal dynamics in phytoplankton community structure or ecosystem health. Given the pronounced seasonal succession of phytoplankton communities, a single sampling event is unlikely to fully characterize species diversity across the study area, and taxa occurring primarily in other seasons or at consistently low abundance may have remained undetected. Therefore, the observed species composition and P‐IBI assessment should be regarded as a period‐specific characterization of the ecological condition of Hulun Lake in June 2023 rather than a comprehensive annual‐scale evaluation. Future studies should incorporate repeated or continuous sampling across spring, summer, autumn, and, where feasible, the ice‐covered period, together with multi‐year monitoring, to evaluate the seasonal robustness of the P‐IBI and improve the comprehensiveness and representativeness of ecological health assessments.

## 5 Conclusion

This study systematically investigated phytoplankton community structure, α‐diversity, phytoplankton index of biotic integrity and its spatial distribution in Hulun Lake. The results revealed that Bacillariophyta and Chlorophyta dominated the phytoplankton community in Hulun Lake. Higher P‐IBI scores were observed in the central and northern regions of the lake, except for the site XHK, whereas relatively lower scores were recorded in the southern region, except for DNHWZ and GZ. These spatial distribution patterns revealed an uneven distribution of biotic integrity across the lake. NH_3_–N and TOC were positively correlated with P‐IBI, whereas T and TN exhibited negative correlations with P‐IBI. The ecological assessment derived from P‐IBI was highly consistent with the WQI‐based assessment, with both approaches identifying southern Hulun Lake as a priority region for enhanced ecological monitoring, targeted pollution mitigation, and habitat restoration.

## Author Contributions

**Qi An:** formal analysis (equal), visualization (equal), writing – original draft (equal), writing – review and editing (equal). **Ruiying Fu:** conceptualization (lead), data curation (lead), formal analysis (supporting), funding acquisition (equal), investigation (lead), methodology (supporting), project administration (equal), resources (equal), software (supporting), validation (lead), visualization (supporting), writing – original draft (lead), writing – review and editing (lead). **Hao Cui:** formal analysis (equal), investigation (equal). **Yuan Wang:** formal analysis (equal), investigation (equal). **Yihan Huo:** formal analysis (equal), investigation (equal). **Yang Bai:** formal analysis (equal), investigation (equal). **Wei Ding:** formal analysis (equal), investigation (equal). **Bowen Xiao:** formal analysis (equal), investigation (equal). **Chengzhen Jia:** formal analysis (equal), investigation (equal). **Huamin Liu:** project administration (equal), resources (equal). **Lixin Wang:** conceptualization (equal), funding acquisition (equal), project administration (equal), resources (equal), writing – review and editing (equal).

## Funding

This work was supported by the National Natural Science Funds, P.R.China (No. 32571847), Natural Science Foundation of Inner Mongolia Autonomous Region (2025QN03060), Guiding Funds of Central Government for Supporting the Development of the Local Science and Technology (2024ZY0128).

## Conflicts of Interest

The authors declare no conflicts of interest.

## Supporting information


**Figure S1:** Rarefaction curve analysis of the environmental DNA metabarcoding data.

**Figure S2:** WQI values across the 13 sampling sites.

**Figure S3:** Boxplots illustrating the core indicators used in the P‐IBI.

**Table S1:** Analytical methods employed for the determination of physicochemical parameters.
**Table S2:** α‐diversity indices across sampling sites.
**Table S3:** Richness across taxonomic levels and sampling sites.
**Table S4:** Scores of core indicators and P‐IBI scores for each sampling site.
**Table S5:** Summary statistics of DCA for phytoplankton community data in Hulun Lake.

## Data Availability

The raw environmental DNA metabarcoding data were deposited in the China National Center for Bioinformatics (CNCB) database under the accession number PRJCA067416.
